# Updating the maize karyotype by chromosome DNA sizing

**DOI:** 10.1371/journal.pone.0190428

**Published:** 2018-01-02

**Authors:** Jéssica Coutinho Silva, Carlos Roberto Carvalho, Wellington Ronildo Clarindo

**Affiliations:** 1 Laboratório de Citogenética e Citometria, Departamento de Biologia Geral, Centro de Ciências Biológicas e da Saúde, Universidade Federal de Viçosa, Viçosa–MG, Brazil; 2 Laboratório de Citogenética, Departamento de Biologia, Campus Alegre, Universidade Federal do Espírito Santo, Alegre–ES, Brazil; Tulane University Health Sciences Center, UNITED STATES

## Abstract

The karyotype is a basic concept regarding the genome, fundamentally described by the number and morphological features of all chromosomes. Chromosome class, centromeric index, intra- and interchromosomal asymmetry index, and constriction localization are important in clinical, systematic and evolutionary approaches. In spite of the advances in karyotype characterization made over the last years, new data about the chromosomes can be generated from quantitative methods, such as image cytometry. Therefore, using *Zea mays* L., this study aimed to update the species’ karyotype by supplementing information on chromosome DNA sizing. After adjustment of the procedures, chromosome morphometry and class as well as knob localization enabled describing the *Z*. *mays* karyotype. In addition, applying image cytometry, DNA sizing was unprecedentedly measured for the arms and satellite of all chromosomes. This way, unambiguous identification of the chromosome pairs, and hence the assembly of 51 karyograms, were only possible after the DNA sizing of each chromosome, their arms and satellite portions. These accurate, quantitative and reproducible data also enabled determining the distribution and variation of DNA content in each chromosome. From this, a correlation between DNA amount and total chromosome length evidenced that the mean DNA content of chromosome 9 was higher than that of chromosome 8. The chromosomal DNA sizing updated the *Z*. *mays* karyotype, providing insights into its dynamic genome with regards to the organization of the ten chromosomes and their respective portions. Considering the results and the relevance of cytogenetics in the current scenario of comparative sequencing and genomics, chromosomal DNA sizing should be incorporated as an additional parameter for karyotype definition. Based on this study, it can be affirmed that cytogenetic approaches go beyond the simple morphological description of chromosomes.

## Introduction

Since Chiarugi’s work in 1933 [1], plant karyograms have been assembled based on morphological features of all chromosomes in a particular complement–the karyotype. Some of these features are commonly derived from the mitotic metaphase: chromosome shape, absolute/relative size of the chromosomes and their arms, and number and size of secondary constrictions. From these data, the centromeric index and chromosome class are determined [2,3], and the intra- and interchromosomal asymmetry indices are measured [4].

Conventional chromosome analyses, which are based on morphometric data, are still widely performed [5,6]. Via banding and in situ hybridization, specific chromosome segments as well as the distribution and size of heterochromatin and euchromatin have been established, mainly in a few crop species, facilitating the identification of homologous pairs and karyogram assembly [7,8].

Since the historical study of McClintock [9], the characterization of the *Zea mays* L., 1753, (Poaceae) karyotype has progressed extensively [10–12]. Over nearly a century of research, *Z*. *mays* has become one of the main plant species in several genetic approaches, due to its intriguing and dynamic genome and crop relevance. Contributing for the significant progress in karyotype understanding, *Z*. *mays* chromosomes have been identified and described via distinct techniques, such as morphometry [9,13], banding [14], fluorescent in situ hybridization [5,15–17], flow karyotyping [18] and image cytometry (ICM) [19]. Knowledge about the karyotype has contributed to taxonomic and systematic studies, and to understand the genome evolution of *Z*. *mays* lines and hybrids [20]. In spite of the approaches conducted in *Z*. *mays*, cytogenetic characterization of this species still presents divergences, especially regarding different cytogenetic classifications for the majority of its ten chromosomes [5,11,15–17,21–23].

In addition to these karyotype discrepancies, disparity in nuclear genome amount among *Z*. *mays* lines have been reported, ranging from 4.62 to 6.29 pg [24,25]. This intraspecific genome size variation possibly reflects karyotype divergences that may have accumulated in different regions of the chromosomes [25,26]. These variations are primarily due to differences in the heterochromatin amount, mainly in knobs [27,28]. Intraspecific DNA amount differences in this species have also been attributed to retrotransposon families, making up over 70% of its nuclear genome [10,29].

Regarding the need for new applications, even in the molecular cytogenetics era, the ICM technique provides quantitative data about the nuclei [30] and chromosomes via DNA sizing [31]. ICM is an accurate, quantitative and reproducible tool based on the analysis of digital images, associating classical cytogenetics and image analysis [32]. The ICM application has allowed to measure the DNA amount of nuclei (in fungi, human, animals and plants) and chromosomes (in a few animal and plant species). Thereby, ICM data have been explored in human clinical studies (mainly cancer diagnosis in distinct tissues) [33,34], for chromosome characterization (in animal and plant species) [19,35], and for understanding the life cycle (in fungi species) [36]. Considering only the chromosomal DNA amount data, ICM has been applied to support investigations on the structure and evolution of chromosomes and genomes through morphology and DNA amount [37,38]. Furthermore, ICM reveals small DNA amount variations in plants with B chromosomes, which can thus be discriminated and individualized in relation to A and other chromosomes [19].

Since the emergence of plant genomic sequencing projects and comparative mapping, karyotype characterization by traditional techniques has become insufficient as input data. Therefore, the aim of this study was to determine the DNA amount in the chromosomes, chromosome arms and satellite of *Z*. *mays*. Based on the obtained results, we propose that chromosomal DNA content determination by ICM should be incorporated as an additional parameter in karyotype characterization.

## Materials and methods

### Plant material

Seeds of commercial *Z*. *mays* ‘AL Bandeirante’ (allotment seed number 87/2014, category 52, harvest 2014/2014, germination rate of 94.0%, 99.8% purity, certified on October 7, 2014) were germinated in Petri dish containing distilled water (dH_2_O) at 30°C. The line ‘AL Bandeirante’ was preferred to other lines owing to its high seed germination rate and metaphase index from its root meristems. *Z*. *mays* ‘CE-777’ seeds were kindly provided by Dr. Jaroslav Doležel (Experimental Institute of Botany, Sokolovská, Czech Republic); leaves of this line were used as internal standard in the flow cytometry (FCM) procedure.

### Nuclear DNA sizing

Leaf fragments (2 cm^2^) from five individuals of ‘AL Bandeirante’ and ‘CE-777’ (2C = 5.57 pg; [39]) were chopped [40] for 30 s in 0.5 mL of OTTO-I lysis buffer [41] supplemented with 2.0 mM dithiothreitol (Sigma^®^) and 50 μg mL^-1^ RNAse (Sigma^®^), pH 2.3. To each nuclei suspension were added 0.5 mL of OTTO-I lysis buffer, and the homogenate was filtered through 30-μm nylon filter (Partec^®^) into 2.0-mL microcentrifuge tubes. The tubes were centrifuged (ALC^®^ MicroCentrifugette^®^ 4214) at 100 ×*g* for 5 min, the supernatant was poured out, and the pellet resuspended and incubated for 5 min in 100 μL of OTTO-I lysis buffer. The suspensions were stained for 20 min with 1.5 mL OTTO-II solution [41] supplemented with 75 μM propidium iodide (PI, Sigma^®^), 2.0 mM dithiothreitol (Sigma^®^) and 50 μg mL^-1^ RNAse, pH = 7.8 [39]. After filtration through 20-μm nylon mesh (Partec^®^), the suspensions were analyzed in a Partec PAS^®^ flow cytometer (Partec^®^ GmbH, Munster, Germany) equipped with a laser source (488 nm). Nuclei-emitted PI fluorescence was detected by a RG 610 nm band-pass filter. Five independent repetitions were performed for each ‘AL Bandeirante’ individual, with over 10,000 nuclei being analyzed each time. The nuclear 2C DNA value was calculated by dividing the mean G_0_/G_1_ fluorescence peak channel of the standard ‘CE-777’ by that of ‘AL Bandeirante’ (sample).

### Cytogenetic preparations

‘AL Bandeirante’ roots of 1-cm length were incubated for 18 h in 0.20 g L^-1^ MS salts [42] and 1.75, 2.00 or 3.00 mM hydroxyurea (HU) (Sigma^®^), at 30°C. The roots were washed in dH_2_O for four times of 15 min at 30°C, then treated with 2–3 μM amiprophos-methyl (APM, Nihon Bayer Agrochem K. K.^®^) for a period of 1–6 h, at 30°C. Next, the roots were fixed three times in solution of methanol and acetic acid 3:1 (v/v) (Merck^®^) for 24 h, followed by three times in 95% ethanol, and stored at -20°C [43].

After washing three times in dH_2_O for a total of 30 min, the roots were macerated in enzyme pool (4% cellulase + 0.4% hemicellulase + 1% pectolyase diluted in pectinase solution, Sigma^®^) diluted in dH_2_O in the proportion 1:8 (enzyme: water). The maceration procedure was performed for 2 h at 35°C. The enzymatic solution was replaced by dH_2_O, the roots were fixed twice in 95% ethanol and stored at -20°C. The slides were prepared using cell dissociation and air-drying technique [43].

### Differential DAPI staining

Some slides, previously chosen based on metaphase number, were treated with 70% formamide/SSC 2X, pH 7.0, at 72°C for 3 min. Next, the slides were exposed to 70%, 85% and 100% ethanol for 5 min each at -20°C. The slides were stained using 5 μM 6-diamidino-2-phenylindole (DAPI) for 5 min, then washed in phosphate buffer saline (PBS) for 3 min. The slides were analyzed under a trinocular epifluorescence microscope Olympus^TM^ BX-60 equipped with WU filter. The images were visualized using an UPlanFI objective with magnification ×100 and 1.4 numeric aperture, and captured using a digital video camera (Olympus^®^ DP71) of 12.5 megapixels and 12-bit color that displays the native CCD’s full-resolution live image at 15 frames per second.

### Chromosomal DNA sizing

For the Feulgen procedure, other slides, also selected based on metaphase number, were immediately placed into 17:5:1 solution of methanol: 37% formaldehyde: acetic acid (Merck^®^) for 12 h at 25°C, washed in dH_2_O for 10 min, air-dried, and hydrolyzed in 5 M HCl (Merck^®^) for 16–20 min at 25°C [43]. After the hydrolysis step, the slides were again washed in dH_2_O for 10 min and air-dried, then stained with Schiff’s reagent (5 g of basic fuchsine, Merck^®^; 15 mL of 1 M HCl; 2.23 g of K_2_S_2_O_5_; 0.703 g of active charcoal, Synth; and 85 mL of dH_2_O; [44]) for 12 h at 4°C. Finally, the slides were washed for three times of 3 min in 0.5% SO_2_-water (Merck^®^) and in dH_2_O.

The setup for ICM analysis and the calibration for microscope procedures were performed according to Carvalho et al. [43]. Briefly, a standard stage micrometer slide (1,000 μm, Olympus^TM^) for the area (spatial) parameter, a neutral density filter (ND6) plus 0.15, 0.30, 0.40, 0.60, 0.90 and 2.50-calibrated density filters for the optical density (OD) parameter, and a linear 11-stepped density filter (Edmund Industrial Optics^®^, Barrington, NJ, USA) for the linearity test were used. The stability [45,46], linearity [43–45,47] and uniformity tests [46,48] were employed to evaluate the setup and calibrations.

Metaphase images were captured using a 12-bit CCD digital video camera (Olympus^®^ DP71) coupled to a trinocular photomicroscope Olympus^TM^ BX-60, equipped with stabilized light source, UPlanFI objective with magnification ×100 and 1.4 numeric aperture, aplanat achromat condenser with 1.4 aperture, neutral density filter (ND6) and interference green color filter (IF 550–570 nm).

Chromosomes of 51 metaphases were digitally segmented using the Image Pro-Plus^®^ 6.1 analysis system (Media Cybernetics^®^). The morphometric data (total length, length of the long and short arms) were used for chromosome class determination according to Levan et al. [2], revised by Guerra [3]. The chromosome ICM was performed using the data on morphometry, area and OD. The integral optical density (IOD) data were automatically measured for each chromosome [43]. The DNA amount of each *Z*. *mays* chromosome was proportionally determined by distributing the nuclear DNA amount (from FCM) by the mean IOD value (from ICM) of each of the 102 sampled chromosomes, chromosome arm and satellite portion. The values were estimated according to the formulas proposed by Carvalho et al. [43]:

$$I:\;2Cn=(\frac{\sum 2Cn}{r})$$

II:IODc=(∑IODpcxn)/2

III:IODt=∑IODc

IV:2Cc=(2CnxIODc)/IODt

where 2C_n_ = nuclear 2C value (pg), *r* = number of FCM repetitions, IOD_c_ = 2C IOD value, IOD_pc_ = chromosomal 2C IOD value, n = number of metaphases, IOD_t_ = IOD value of all chromosomes, and 2C_c_ = chromosomal 2C DNA amount.

The DNA amount of each chromosome arm and of the satellite portion was measured by the equations:

$$I:2Cn=(\frac{\sum 2Cn}{r})$$

II:IODb=(∑IODbcxn)/2

III:IODt=∑IODc

IV:2Cb=(1CnxIODb)/IODt

where 2Cn = nuclear 2C value (pg), r = number of FCM repetitions, IOD_b_ = 2C IOD value of the chromosome arm, IOD_bc_ = 2C IOD value of the chromosome short arm, *n* = number of metaphases, IOD_t_ = IOD value of all chromosome, and 2C_b_ = 2C DNA amount of the chromosome arm or satellite portion.

The mean value of chromosomal 2C amount was converted to 1C to be made comparable to values reported by other studies [18, 19] and genomic sequencing (http://ensembl.gramene.org/Zea_mays/Location/Genome?r=1:8001-18000). In order to enhance the reproducibility, the ICM protocol for chromosomal DNA sizing was deposited in protocols.io. The same protocol can be adapted for other species and for nuclear DNA sizing.

## Results

### Nuclear DNA sizing

**Fig 1:**
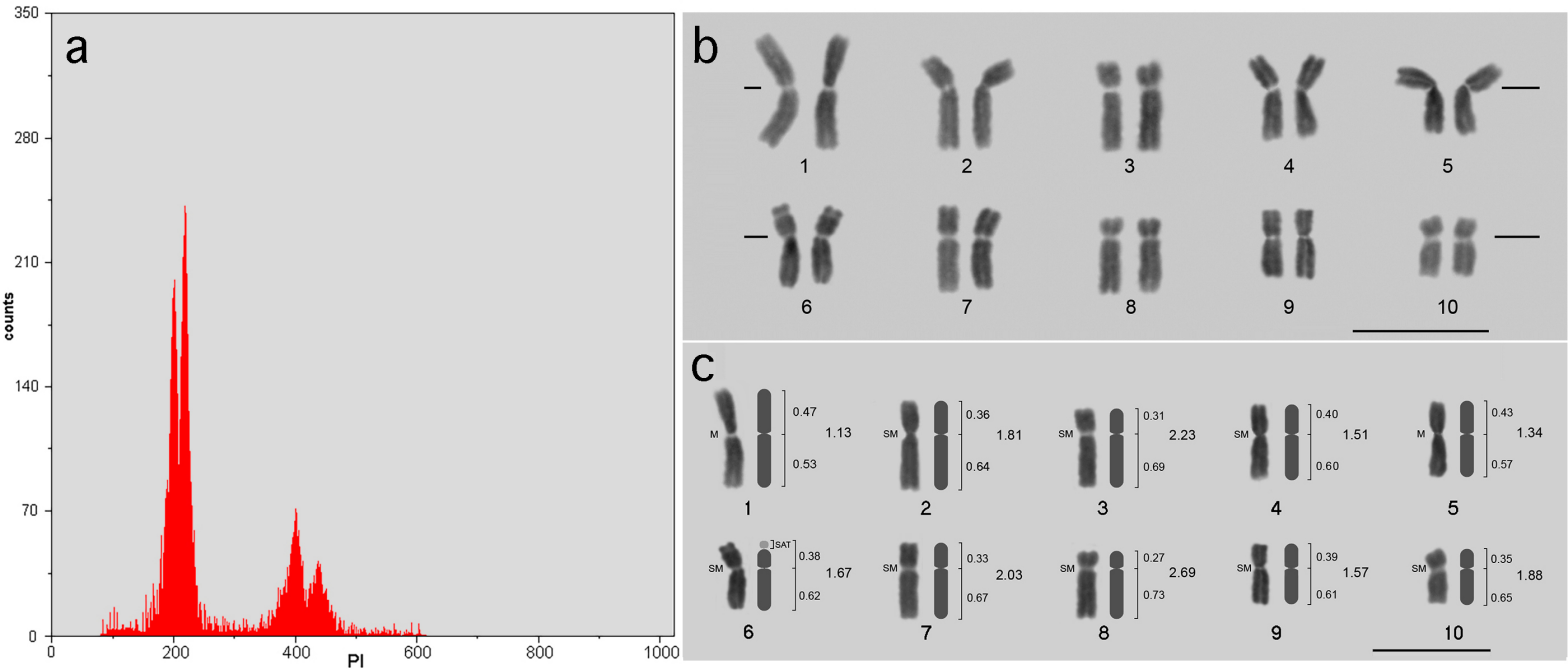
Representative histogram and karyogram of *Z*. *mays*. **(A)** Representative histogram from FCM measurement of the nuclear DNA amount of *Z*. *mays* ‘AL Bandeirante’ from suspensions stained with propidium iodide. Channel 200 shows the G_0_/G_1_ peak of ‘CE-777’ (internal standard), 2C = 5.57 pg and CV = 3.20%, and channel 219 the G_0_/G_1_ peak of sample ‘AL Bandeirante’, 2C = 6.10 pg and CV = 3.80%. **(B)** Representative karyogram of *Z*. *mays*. Prometaphase obtained from root meristem treated with 1.75 mM HU for 18 h and 3 μM APM for 4 h, followed by Feulgen reaction. The further preparations display stoichiometrically stained chromosomes with well-defined telomeric, centromeric and secondary constriction portions, which are prerequisites for morphometric characterization and determination of area, OD and IOD. Bar = 10 μm. **(C)** Ideogram assembled based on chromosome morphometry. Relative arm length and arm ratio are respectively shown to the right of the chromosome representation. Bar = 10 μm.

*Z*. *mays* ‘AL Bandeirante’ showed mean 2C = 6.10 ± 0.044 pg, corresponding to 2C = 5.96 × 10^9^ bp. Considering the high resolution of the G_0_/G_1_ peaks, revealed by coefficients of variation (CV) between 3.00% and 4.10%, the 2C nuclear DNA amount of ‘AL Bandeirante’ is 0.53 pg (9.52%) larger than that of ‘CE-777’. Therefore, FCM evidenced an intraspecific variation between the two *Z*. *mays* lines (Fig 1A).

### Cytogenetic preparations

The cell cycle was reversibly inhibited in S phase by the root treatment with 1.75 mM HU for 18 h. From these roots, several metaphases were obtained using 3 μM APM for 4 h. Preliminary tests revealed a metaphase index of 0.80% in untreated root meristems, which is very inferior in relation to meristems exposed to HU (47%) and HU followed by APM (61%).

Fixation, enzymatic root maceration and slide preparation were also crucial. These procedures provided cytoplasm-free metaphases with morphologically preserved and isolated chromosomes exhibiting well-defined telomere and primary and secondary constrictions. These features were fundamental to establish the chromosome morphometry, area and OD values, and to assemble the 51 karyograms (Fig 1B) and one representative ideogram (Fig 1C).

### Differential DAPI staining

Higher fluorescence intensity of DAPI was verified in knobs located in interstitial chromosome portions of the long arms of chromosomes 2–9. Thus, the differential DAPI staining also facilitated the identification of chromosome pairs and karyogram assembly (S1 Fig).

### Chromosomal DNA sizing

The chromosomes were stoichiometrically stained by Feulgen procedure including 5 M HCl for 18 min at 25°C and Schiff’s reagent for 12 h at 4°C (Fig 1B). The depurination step with HCl also contributed to remove residual cytoplasmic background and to the staining by Schiff’s reaction during the Feulgen procedure. As a result, the maize chromosomes presented well-defined telomeric portions as well as centromeric and secondary constrictions, which are prerequisites for the unprecedented measurement of the OD (S2 Fig), area (S3 Fig) and, consequently, IOD (S4 Fig) and chromosomal DNA amount (Table 1). All 51 karyograms showed 2n = 2x = 20 chromosomes, being two metacentric (1 and 5), eight submetacentric (2–4, 6–10), and one pair (6) exhibiting the nucleolar organizer region (NOR) in the terminal portion of the short arm (Fig 1C).

**Table 1:** Mean 2C DNA value of each maize chromosome arm and satellite portions in pg (± standard deviation) and in 1C bp.

Chromosome	Arm	2C DNA amount by chromosome arm (pg)	1C Value (bp × 10^9^)^a^
1	S	0.376 ± 0.027	0.184
	L	0.427 ± 0.017	0.209
2	S	0.269 ± 0.023	0.132
	L	0.456 ± 0.030	0.223
3	S	0.237 ± 0.024	0.116
	L	0.440 ± 0.040	0.215
4	S	0.239 ± 0.031	0.117
	L	0.395 ± 0.040	0.193
5	S	0.271 ± 0.019	0.133
	L	0.355 ± 0.020	0.174
6	SAT	0.053 ± 0.009	0.026
	S	0.171 ± 0.021	0.084
	L	0.366 ± 0.035	0.179
7	S	0.156 ± 0.023	0.076
	L	0.408 ± 0.026	0.200
8	S	0.131 ± 0.013	0.064
	L	0.402 ±0.030	0.197
9	S	0.215 ± 0.025	0.105
	L	0.346 ± 0.028	0.169
10	S	0.131 ± 0.008	0.064
	L	0.255 ± 0.013	0.125
Total		6.10	2.983

SAT–satellite; S–short arm; L–long arm.

^a^Values converted to bp, whereby 1 pg of DNA corresponds to 0.978 × 10^9^ bp [49].

In the optical setup, the signal of OD mean values was considered stable after 12 min. Hence, all metaphases were only captured after this time. All other setups for microscope calibration and digital image analysis system were evaluated. The image analysis software calculated a R^2^ = 0.999 for the linearity test and a CV below 3.0% for the uniformity test. For the first time, the area and OD values were thus measured for all chromosomes (total length, short and long arms, satellite) of the 51 karyograms of a plant species.

Mean area values ranged from 9.478 μm^2^ (chromosome 1) to 4.652 μm^2^ (chromosome 10). Chromosome 9 had a mean area (6.672 μm^2^) greater than that of chromosome 8 (6.342 μm^2^). Mean values of OD varied from 1.127 (chromosome 1) to 1.112 (chromosome 5), with the mean values for chromosomes 4 and 5 (1.112) being smaller than for chromosomes 6–10 (1.133 to 1.117). Chromosome 6 had a mean OD value (1.113) smaller than that of chromosome 7 (1.114), and the mean OD value for these chromosomes was smaller than for chromosomes 8–10 (1.117) (S2 and S3 Figs).

**Fig 2:**
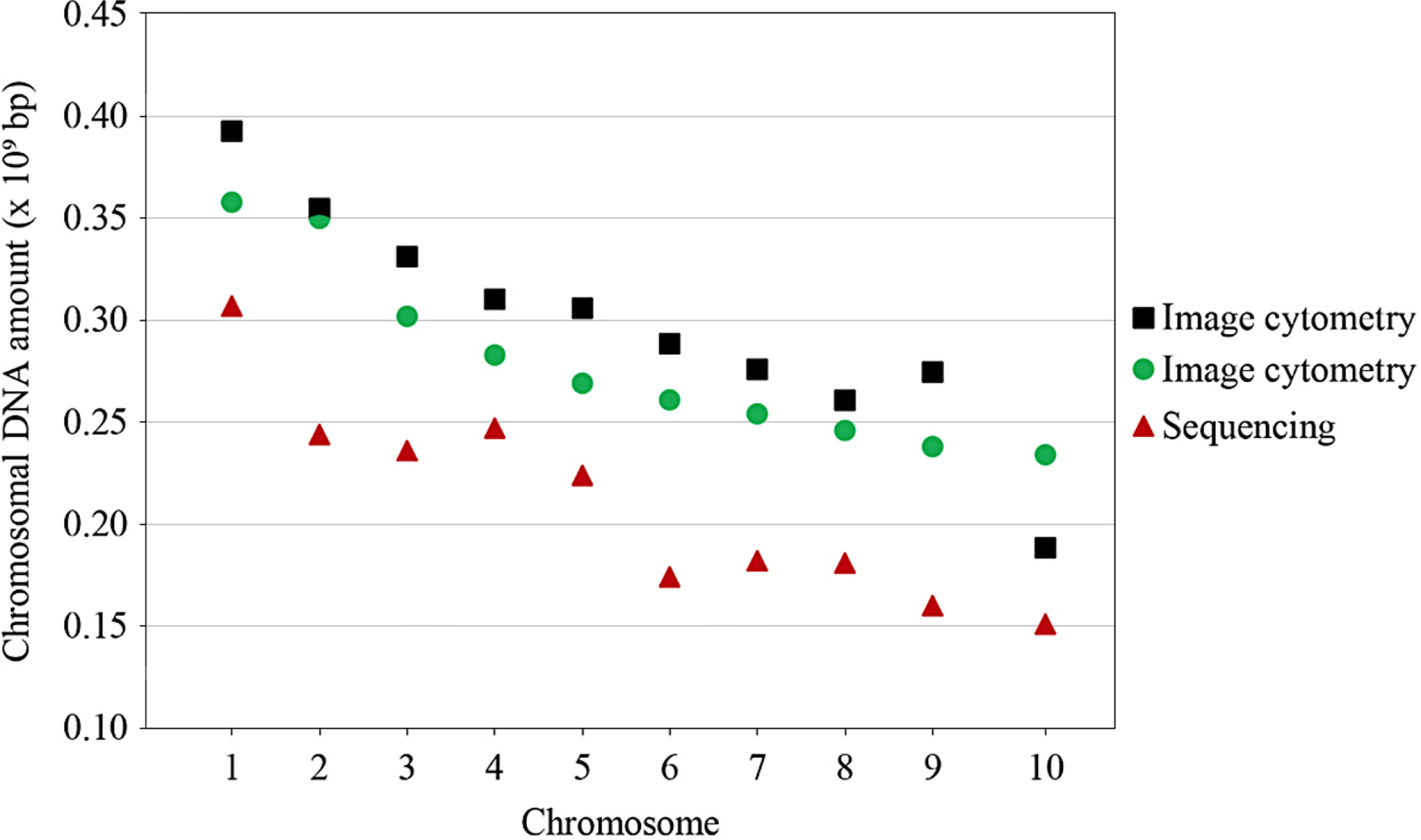
Comparison between chromosomal 1C DNA amount (in bp) of the present study (ICM, *Z*. *mays* ‘AL Bandeirante’; black square), sequencing data (*Z*. *mays* ‘B73’; red triangle) (http://ensembl.gramene.org/Zea_mays/Location/Genome?r=1:8001-18000) and ICM data (*Z*. *mays* ‘Black Mexican Sweet Corn’; green circle) [19]. Note that all values measured for ‘Black Mexican Sweet Corn’, except for chromosome 10 (1C = 0.234 × 10^9^ bp), and by sequencing were lower when compared to ‘AL Bandeirante’. Sequencing values ranged from 1C = 0.301 × 10^9^ bp (chromosome 1) to 1C = 0.150 × 10^9^ bp (chromosome 10). Chromosomal DNA values for ‘AL Bandeirante’ ranged from 1C = 0.393 × 10^9^ bp (chromosome 1) to 1C = 0.188 × 10^9^ bp (chromosome 10). Values converted to bp, considering that 1 pg of DNA corresponds to 0.978 × 10^9^ [49].

Based on area and OD values of each chromosome (total length, short and long arms, satellite), the IOD value was automatically calculated (S2 and S4 Figs). This way, the mean DNA amount could be determined for all chromosome portions based on the distribution of the mean nuclear 2C value (FCM) of ‘AL Bandeirante’ in relation to the mean IOD values (Table 1). Mean 2C values for the short chromosome arm ranged from 0.376 ± 0.027 pg for chromosome 1 to 0.131 ± 0.008 pg for chromosome 10, whereas the 2C values for the long chromosome arm ranged from 0.427 ± 0.017 pg for chromosome 1 to 0.255 ± 0.013 pg for chromosome 10. The mean DNA amount of the satellite portion of chromosome 6 was 2C = 0.053 ± 0.009 pg. Standard deviations (± SD) regarding the DNA amount values varied from 0.008 to 0.040, being that the highest values were associated with the long arms of chromosomes 2–9 (Table 1), which possess knobs (S1 Fig). Corroborating the OD and area data (S2 and S3 Figs), the mean DNA amount of chromosome 9 (2C = 0.561 pg) was higher than that of chromosome 8 (2C = 0.533 pg) (Table 1, Fig 2).

Mean values of the chromosomal 2C DNA amount were converted to 1C × 10^9^ bp for comparability to other values. Differences were verified between the mean values (1C bp) reported here and by Lee et al. [18], Rosado et al. [19], and genomic sequencing (http://ensembl.gramene.org/Zea_mays/Location/Genome?r=1:8001-18000). The mean chromosomal DNA amount of chromosome 1 (1C = 0.365 × 10^9^ bp) reported by Lee et al. [18], which was measured using flow karyotyping, was 7.67% smaller than the value assigned to chromosome 1 in this study (1C = 0.393 × 10^9^). The DNA amount values for all chromosomes of *Z*. *mays* ‘Black Mexican Sweet Corn’ obtained via ICM by Rosado et al. [19] were also lower than the mean values found here, except for chromosome 10. The mean DNA amount values for the ‘AL Bandeirante’ chromosomes were also higher than those obtained via genomic sequencing (http://ensembl.gramene.org/Zea_mays/Location/Genome?r=1:8001-18000), exhibiting 26.00% (chromosome 10) to 74.52% more DNA (chromosome 9) (Table 1, Fig 2).

## Discussion

Meticulous cytogenetic procedures provided a high rate of clear metaphases, exhibiting chromosomes well spread on the slide, without cytoplasmic background or chromatin breakage. These cytogenetic aspects allowed the stoichiometric staining by DAPI and Schiff’s reaction, being considered crucial for chromosome morphometry and DNA amount determination [19,43]. The image analysis system, which was calibrated and evaluated according to medical criteria [43,44,47], also resulted in accurate ICM parameters: area, OD and, thus, IOD. Therefore, these steps were considered essential to obtain reliable, reproducible and quantitative data for all chromosomes of the 51 metaphases.

Chromosomal DNA sizing promoted an update of the *Z*. *mays* karyotype, further allowing to resolve the morphological similarity between chromosomes 2–4 and 7–9 [5]. This way, the 51 karyograms were assembled after identification and classification of all chromosome pairs based on chromosome class (Fig 1B and 1C), DAPI fluorescence bands (S1 Fig), and especially chromosomal DNA amount (Table 1, Fig 2). Attempts to resolve the *Z*. *mays* karyotype have been made according to rules proposed by Chiaruhi [1] for karyogram assembly. However, controversies regarding chromosome class have been reported, for instance: three metacentric (1, 2 and 5), six submetacentric (3, 4, 6, 7, 9 and 10) and one acrocentric (8) chromosome pair in ‘KYS’ [16] and ‘Black Mexican Sweet Corn’ [19]; six metacentric (1, 2, 3, 4, 5 and 9) and four submetacentric pairs (6, 7, 8 and 10) in ‘Amarillo Chico’ [28]; and two metacentric (1 and 5) and eight submetacentric pairs (2–4, 6–10) in the present study (Fig 1C). Thus, varying chromosome classes have been determined for *Z*. *mays* chromosomes 2, 3, 4, 8 and 9. In spite of changes promoted by chromosome rearrangements [17], the polymorphism of the knobs has been appointed as the main cause of the karyotype variation in *Z*. *mays*, affecting 5–20% of the chromosome arm length [5]. Although interfering with chromosome structure, the quantitative chromosomal DNA sizing, in association with the further parameters, allowed the unambiguous identification of each chromosome pair, increasing the resolution for karyogram assembly.

The variations observed in the area, OD (S2 and S3 Figs) and DNA amount of the maize chromosomes (Table 1) may be caused by knob polymorphism, which has been reported for distinct lines [5,17,20]. This hypothesis is corroborated by the DNA amount of chromosome 9 being larger than that of chromosome 8 (Table 1, Fig 2), and the differential DAPI staining evidencing a knob portion in the long arm of chromosome 9 (S1 Fig). Mondin et al. [17] demonstrated that these divergences could be the result of different amounts of repetitive DNA.

Comparing the chromosomal DNA amounts of ‘AL Bandeirante’ (2C = 6.10 pg) and ‘Black Mexican Sweet Corn’ without B chromosomes (2C = 5.72 pg, [19]), significant differences were found in the chromosomes displaying knobs. These portions were confirmed by differential DAPI fluorescence in the long arms of chromosomes 2–9 (S1 Fig). Besides, the highest standard deviation values were obtained for the chromosomal DNA amount of long arms containing knob portions (chromosomes 2–9, S1 Fig, Table 1). Regarding these, the data suggest that the intraspecific variation in nuclear and chromosomal DNA amount was promoted by differential heterochromatin amounts in knobs, as also suggested by previous studies [27,28]. The activity of retrotransposon families, which make up over 75% of the *Z*. *mays* genome [10,50], is appointed as the main phenomenon that culminates in the DNA amount fluctuations detected in this study. In *Z*. *mays*, retrotransposons are abundant in the knob regions [51]. These regions have been observed in 34 distinct regions of *Z*. *mays* chromosomes, varying in size and number among distinct lines [52]. Knobs behave as megatransposons, owing to the presence of different types of retrotransposable elements, which may transpose the knobs from one region to another [53]. Other factors, such as meiosis, also influence the polymorphism in these regions by recombination [54].

The chromosome 1 of ‘AL Bandeirante’ showed 9.69% higher DNA amount (2C = 0.803 pg) compared to ‘Black Mexican Sweet Corn’ (2C = 0.732 pg) (Fig 2), supporting the nuclear DNA amount of this line (2C = 6.10 pg) (Fig 1A). Differently from the other chromosomes (2–9), the knob portion was not differentiated by DAPI staining in this chromosome (S1 Fig). Knob portions have been reported in the arms of the maize chromosome 1, with the knob of the short arm being found in more than 50% of the lines [22]. Considering this, the present data reinforce the knob polymorphism in maize. Realini et al. [25] reported that other non-coding, repetitive DNA sequences contribute for the genome size variation in *Z*. *mays*. Furthermore, repetitive DNA sequences distributed throughout the genome are a major component of eukaryotic genomes and may account for up to 90% of their size [55].

Besides updating the *Z*. *mays* karyotype, the present ICM data should be added to the Maize Genetics and Genomics Database, evidencing the total complexity of the genome and providing data for comparison to other species and lines. Here, the chromosomal DNA amount was higher in all chromosomes compared to sequencing data (Fig 2). This result may reflect an excessive DNA amount in ‘AL Bandeirante’ (Fig 1A) or an underestimation of the bp number in sequencing. The genome of *Z*. *mays* has a high amount of repetitive DNA [10], which is often not considered in the sequencing-based assembly [26]. As shown here, the knowledge conferred by ICM on the genome complexity via resolution of chromosome arms and satellite allowed verifying the variations in DNA amount among chromosomes.

The *Z*. *mays* karyotype has been studied since the work by McClintock [9], representing a model in plant cytogenetics. As in other species, the hindrances related to cytogenetic procedures, karyotype similarities and changes in *Z*. *mays* require a constant search for new applications to characterize its chromosomes. Hence, in this work chromosomal DNA sizing resolved the *Z*. *mays* karyotype, updating the description of all chromosomes and their respective portions: arms of all chromosomes and satellite in chromosome 6. The same quantitative data, associated to classical cytogenetics and DAPI staining, allowed determining the genome size variation and its distribution in the chromosomes and their respective arms. Therefore, we hereby suggest that chromosomal DNA sizing should be incorporated for karyotype description in plants.

## Supporting information

S1 Fig Karyogram showing positive DAPI regions, corresponding to the knobs located in the long arms of chromosomes 2–9. The knobs were identified according to the cytological map of *Z*. *mays* chromosomes [22]. Note the secondary constriction in the short arm of chromosome 6. Bar = 10 μm.
(TIF)

S2 Fig Mean OD values of *Z*. *mays* chromosomes generated from 102 chromosomes (51 karyograms). The OD of the chromosomes ranged from 1.127 (chromosome 1) to 1.112 (chromosome 5). Note that the density of chromosomes 4 and 5 (1.112) is lower than that of chromosomes 6–10 (1.133–1.117); the density of chromosome 6 (1.113) is lower than that of chromosome 7 (1.114); and that the OD of chromosomes 6 and 7 is smaller than for chromosomes 8–10 (1.117).
(TIF)

S3 Fig Mean values for area of each *Z*. *mays* chromosome, from measurement of 102 chromosomes (51 karyograms). The median chromosome areas ranged from 9.478 (chromosome 1) to 4.652 μm^2^ (chromosome 10). Note that the area of chromosome 9 (6.672 μm^2^) is greater in relation to chromosome 8 (6.342 μm^2^).
(TIF)

S4 Fig Mean IOD values of *Z*. *mays* chromosomes measured from the ratio between area and OD values. IOD values ranged from 10.679 (chromosome 1) to 5.196 (chromosome 10). Note that the IOD of chromosome 9 (7.454) is higher than that of chromosome 8 (7.083).
(TIF)
