# Supplementary figures and images for: Updating the maize karyotype by chromosome DNA sizing
Source: PLoS One. 2018 Jan 2;13(1):e0190428. doi: 10.1371/journal.pone.0190428 (PMC5749775; doi:10.1371/journal.pone.0190428)

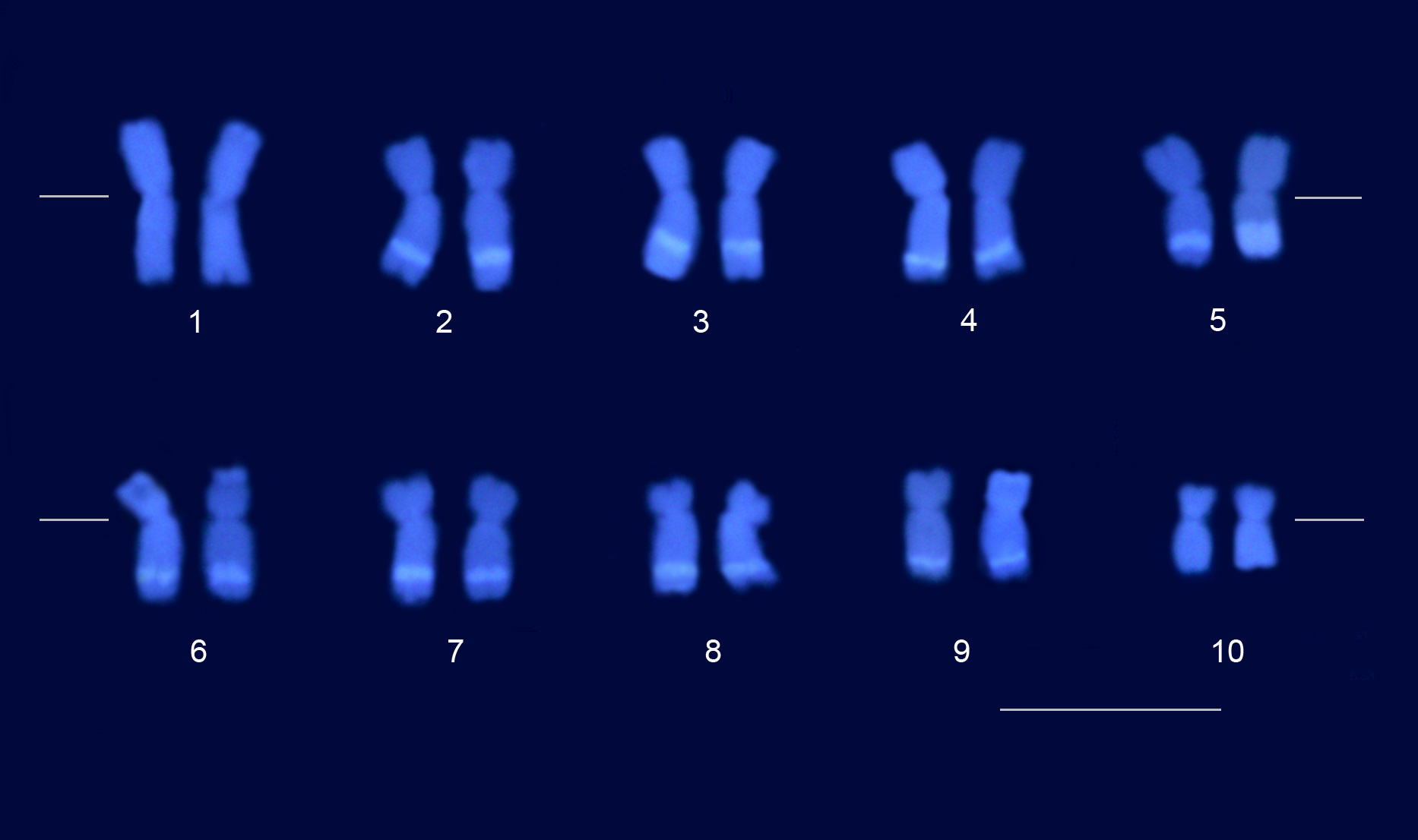

Supplement: S1 Fig — The knobs were identified according to the cytological map of Z. mays chromosomes [22]. Note the secondary constriction in the short arm of chromosome 6. Bar = 10 μm. (TIF) [file pone.0190428.s001.tif]

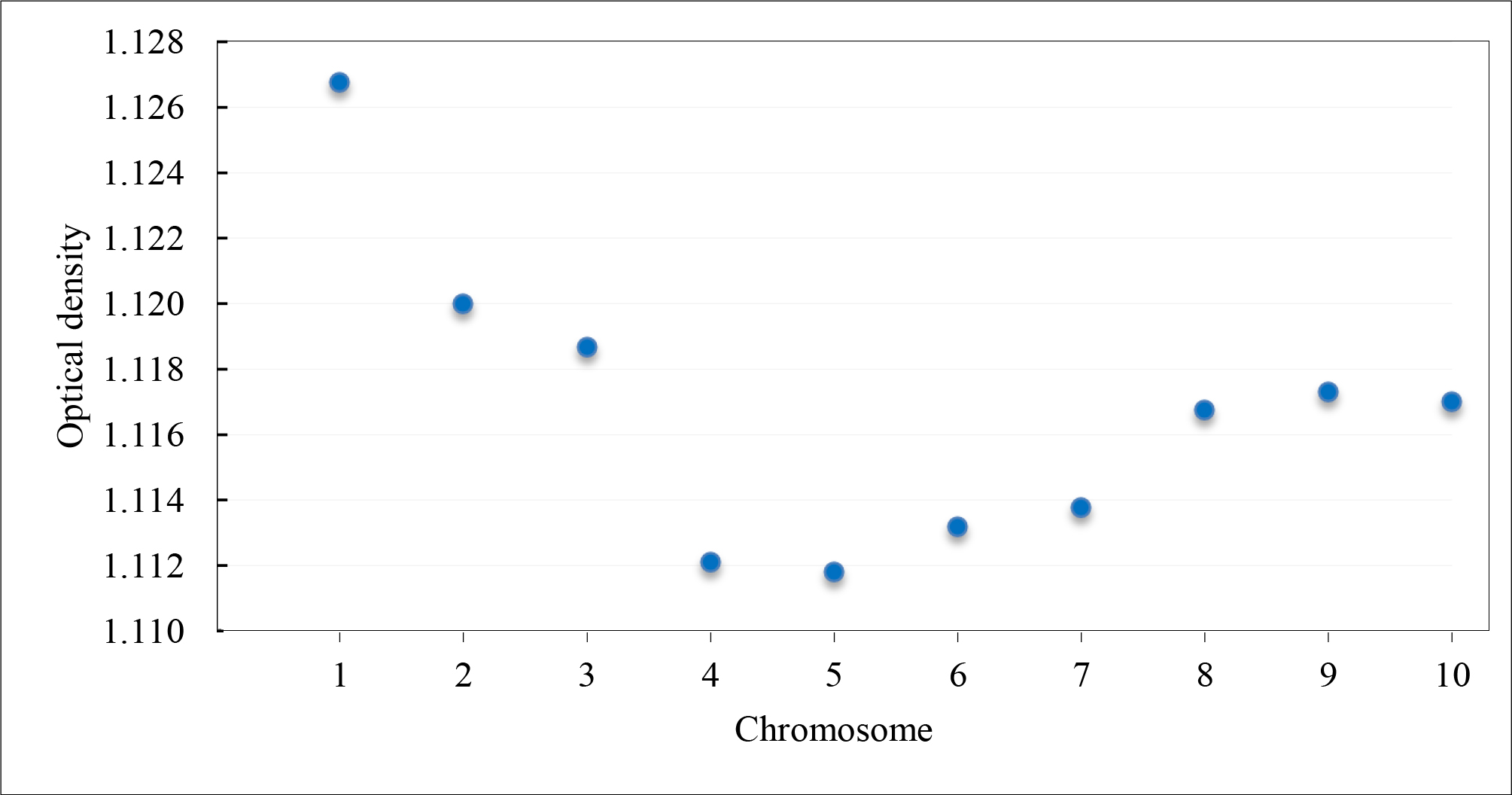

Supplement: S2 Fig — The OD of the chromosomes ranged from 1.127 (chromosome 1) to 1.112 (chromosome 5). Note that the density of chromosomes 4 and 5 (1.112) is lower than that of chromosomes 6–10 (1.133–1.117); the density of chromosome 6 (1.113) is lower than that of chromosome 7 (1.114); and that the OD of chromosomes 6 and 7 is smaller than for chromosomes 8–10 (1.117). (TIF) [file pone.0190428.s002.tif]

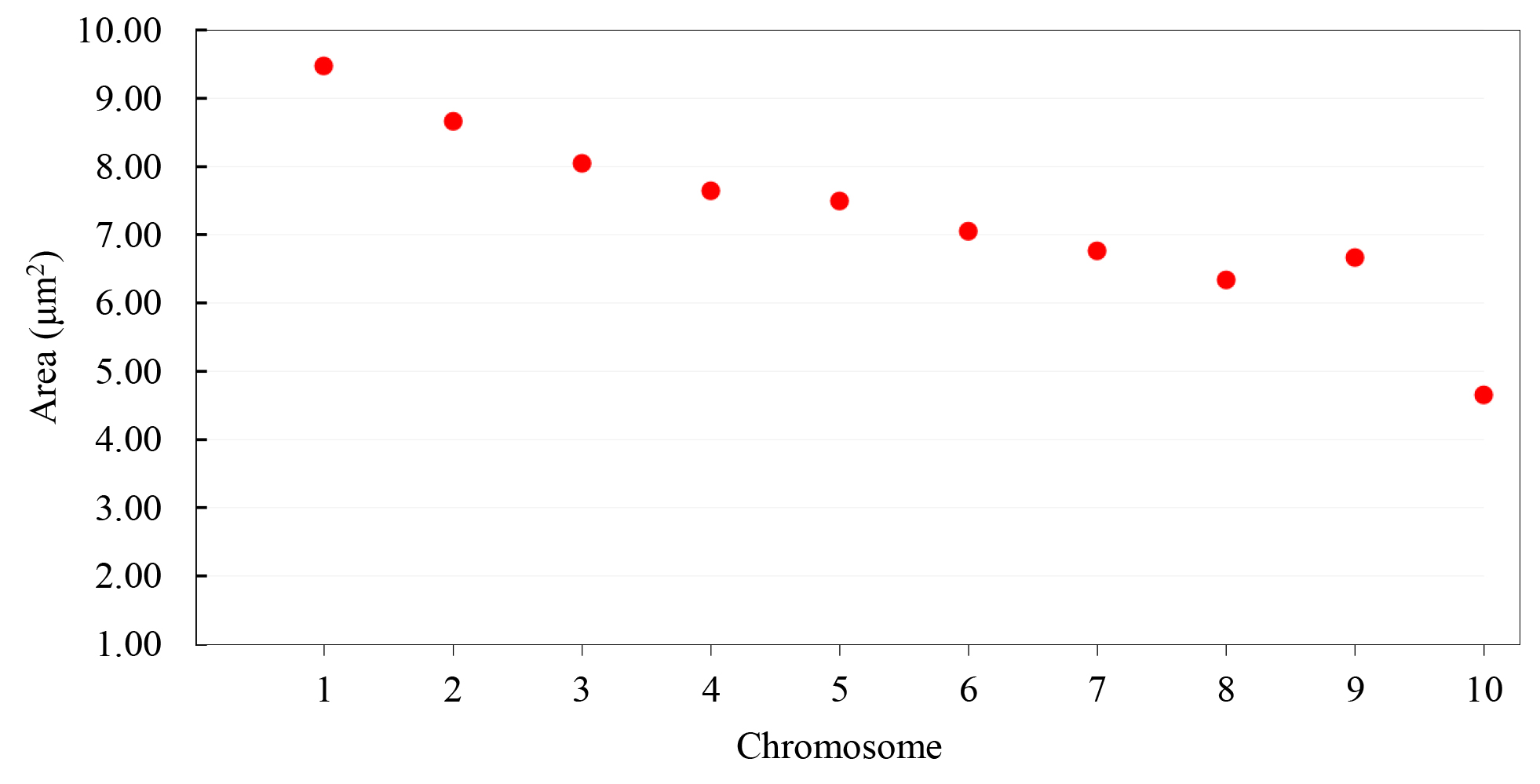

Supplement: S3 Fig — The median chromosome areas ranged from 9.478 (chromosome 1) to 4.652 μm2 (chromosome 10). Note that the area of chromosome 9 (6.672 μm2) is greater in relation to chromosome 8 (6.342 μm2). (TIF) [file pone.0190428.s003.tif]

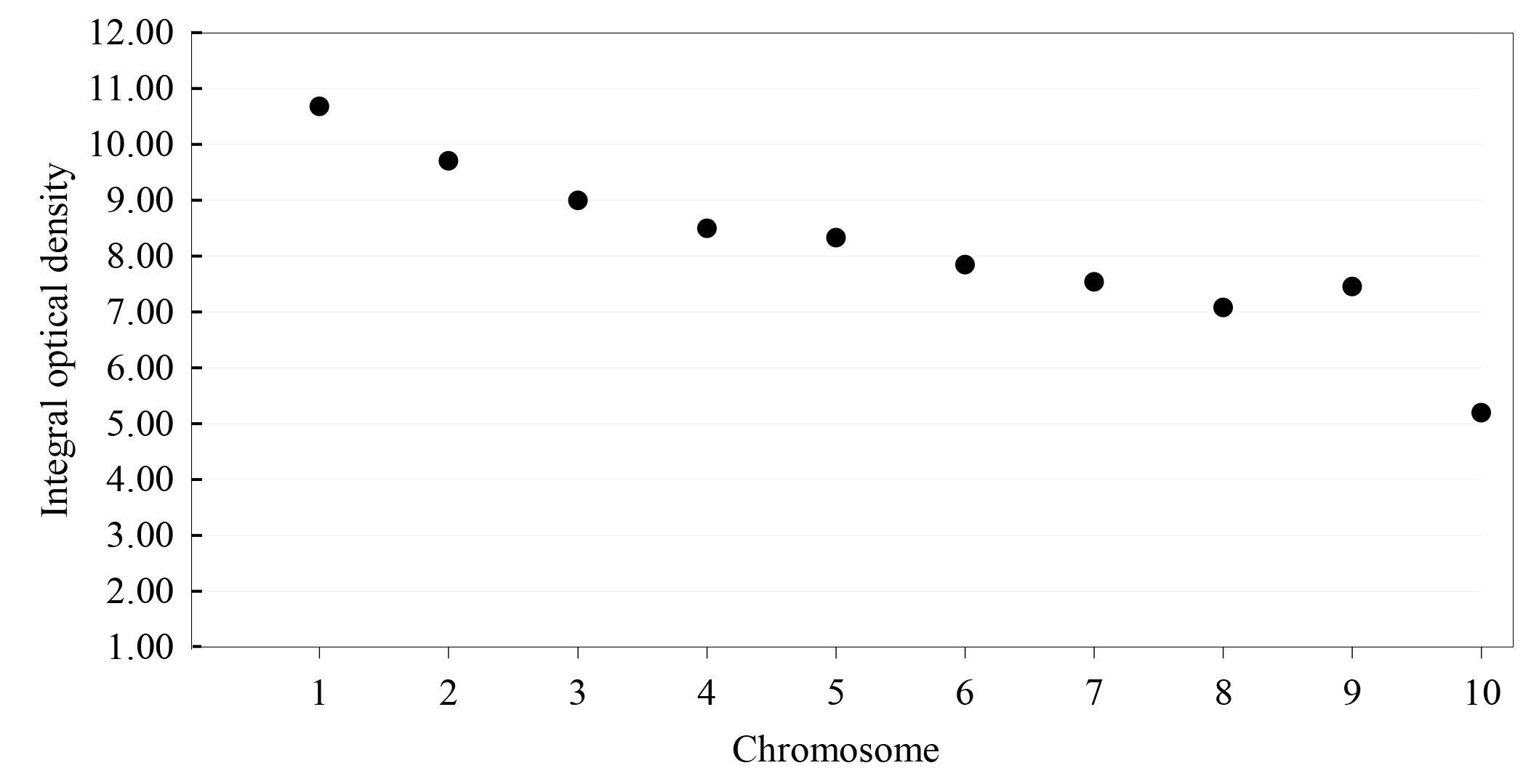

Supplement: S4 Fig — IOD values ranged from 10.679 (chromosome 1) to 5.196 (chromosome 10). Note that the IOD of chromosome 9 (7.454) is higher than that of chromosome 8 (7.083). (TIF) [file pone.0190428.s004.tif]
